# Supplementary material for: Skeletal Muscle 31P Magnetic Resonance Spectroscopy Study of Patients with Parkinson’s Disease: Energy Metabolism and Exercise Performance
Source: Diagnostics (Basel). 2025 Oct 13;15(20):2573. doi: 10.3390/diagnostics15202573 (PMC12562816; doi:10.3390/diagnostics15202573)
Supplement: Supplementary file 1 [file diagnostics-15-02573-s001.zip › diagnostics-3794679-supplementary.pdf]

Supplementary Materials

Skeletal Muscle ^{31}P Magnetic Resonance Spectroscopy Study of Patients with Parkinson's Disease: Energy Metabolism and Exercise Performance

Jimin Ren [1,2*], Neha Patel [1], Talon Johnson [1], Ross Querry [3] and Staci Shearin [3,*]

[1] Advanced Imaging Research Center, University of Texas Southwestern Medical Center, Dallas, TX 75390, United States
[2] Department of Radiology, University of Texas Southwestern Medical Center, Dallas, TX 75390, United States
[3] Department of Physical Therapy, University of Texas Southwestern Medical Center, Dallas, TX 75390, United States

* Authors to whom correspondence should be addressed(J.R. jimin.ren@utsouthwestern.edu; S.S. staci.shearin@utsouthwestern.edu)

Figure S1. Photographs showing (A) the experimental setup of the ^{31}P/^{1}H dual-tuned RF coil and the custom-built plantar flexion ergometer positioned on the scanner table of a 7T MRI system. **(B)** Front view and **(C)** side view of the exercise device, with key components labeled:

1. High-strength pulley cable
2. Cable guard
3. Roller
4. Pedal
5. Rear stoppers
6. Adjustable stopper base board (slides forward/backward)
7. Device base board
8. Foot pad (attached to pedal for force application)
9. Straps for stabilizing the ankle (9a), calf (9b), and knee (9c)
10. ^{1}H/^{31}P dual-tuned RF coil
11. ^{1}H transmit/receive cables
12. ^{31}P transmit/receive cables

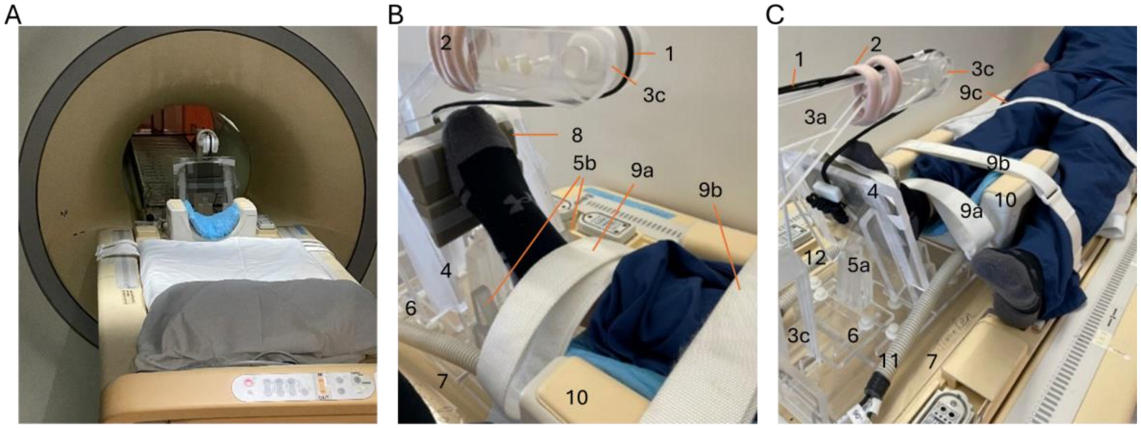

Figure S2. Curves of relative Z-magnetization versus inversion-recovery time (TI) for the 5-spin system in the three-module mEBIT experiment.
(A) Module I: inversion of Pi and PCr.
(B) Module II: inversion of α-, β-, and γ-ATP.
(C) Module III: inversion of PCr and α-, β-, and γ-ATP.

Solid lines represent fitted data (from Figure 4), and dashed lines represent simulated data, modeled with (solid) and without (dashed) chemical exchange and nuclear Overhauser effect (NOE) between neighboring ATP spins.

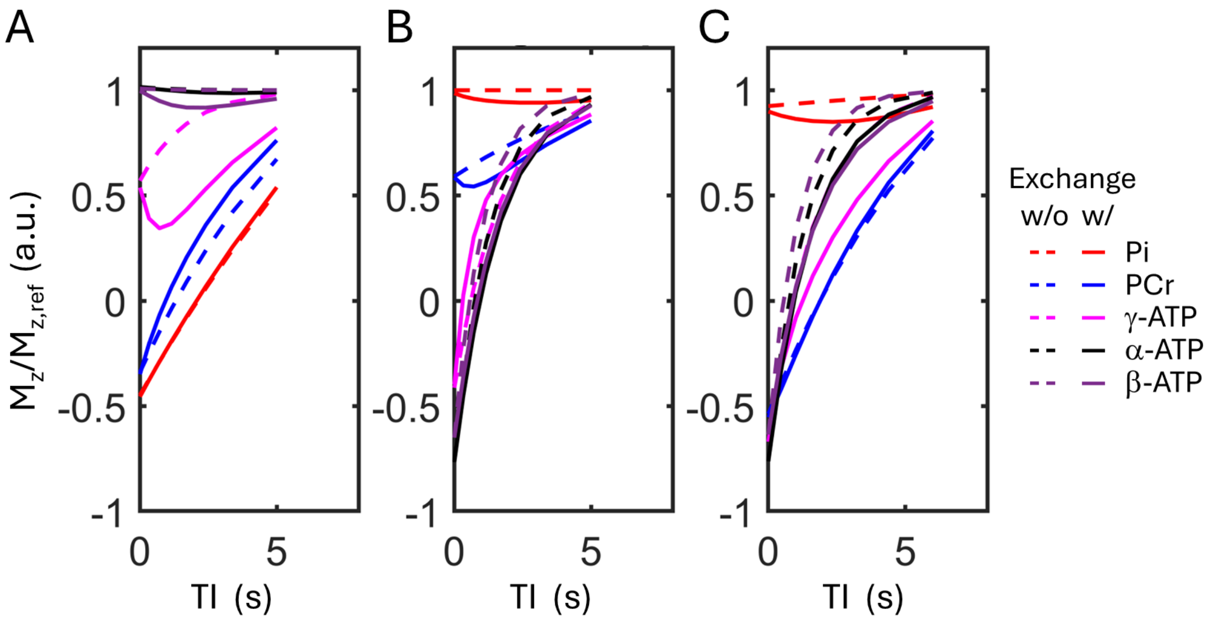

Table S1. Results of ^{31}P intrinsic T_1 relaxation time and ATP ^{31}P-^{31}P cross-relaxation rate (σ) from analysis of kinetic MRS data at 7T

	PD		Control		p-val		
	**pre**	**post**	**pre**	**post**	**PD[1]**	**ctrl[1]**	**PD-vs-ctrl[2]**
σ, s^{-1}	-0.23 (0.10)	-0.29 (0.15)	-0.39 (0.13)	-0.34 (0.06)	0.20	0.42	0.04*
T_1, s							
PCr	6.4 (1.3)	6.5 (1.3)	6.8 (0.9)	7.1 (1.0)	0.93	0.39	0.26
Pi	11.8 (3.6)	11.1 (3.5)	9.9 (1.6)	9.9 (1.2)	0.16	0.39	0.28
γ-ATP	1.7 (0.4)	1.5 (0.4)	1.4 (0.2)	1.1 (0.3)	0.94	0.42	0.01*
β-ATP	1.0 (0.2)	1.0 (0.2)	0.8 (0.1)	0.8 (0.1)	0.05	0.30	0.01*
α-ATP	1.3 (0.2)	1.3 (0.2)	1.1 (0.1)	1.1 (0.1)	0.60	0.84	0.01*

[1] ttest for differences in measurement between pre- and post-exercise training. [2] ttest for differences in measurement between PD and controls, with measurements averaged over pre- and post-rehab.
